# Measuring transcutaneous bilirubin: a comparative analysis of three devices on a multiracial population

**DOI:** 10.1186/1471-2431-12-70

**Published:** 2012-06-14

**Authors:** Francesco Raimondi, Silvia Lama, Francesca Landolfo, Maria Sellitto, Angela Carla Borrelli, Rosalba Maffucci, Paola Milite, Letizia Capasso

**Affiliations:** 1 Division of Neonatology, Department of Pediatrics, Università “Federico II”, Via Pansini 5, 80131, Napoli, Italy

## Abstract

**Background:**

Hyperbilirubinemia can lead to potentially irreversible bilirubin-induced neurotoxicity. Transcutaneous bilirubin (TcB) determination has become a valuable aid in non invasive screening of neonatal jaundice.

The aim of this study is to compare the performance of three most widespread transcutaneous bilirubinometers on a multiracial population of term and late pre-term neonates.

**Methods:**

Bilirubin concentration was determined using traditional photometric determination and transcutaneously with Bilicheck, BiliMed and JM-103, in random order.

Total serum bilirubin (TSB) was determined over a wide concentration range (15,8–0,7 mg/dl) with a mean of 9,5 mg/dl. Related TcB values using Bilicheck (TcB-BC), BiliMed (TcB-BM), and JM-103 (TcB-JM) are reported in Table 1.

**Results:**

A multiracial population of 289 neonates was enrolled with a gestational age ranging from 35 to 41 weeks; birth weight ranging from 1800to 4350 grams; hours of life ranging from 4 to 424. In the total study population correlation analysis using Pearson coefficients showed good results for Bilicheck (r = 0.86) and JM-103 (r = 0.85) but poor for BiliMed (r = 0,70). Similar results were found for the non-Caucasian neonates subgroup. Bilicheck and JM-103 had a greater area under the curve than BiliMed when TSB =14 mg/dl was chosen as a threshold value both for the total study population and the non-Caucasian subgroup.

**Conclusions:**

Bilicheck and JM-103, but not BiliMed, are equally reliable screening tools for hyperbilirubinemia in our multiracial neonatal population.

## Background

Transcutaneous bilirubin (TcB) determination has become a valuable aid in avoiding significant neonatal hyperbilirubinemia [1] and has significantly reduced the number of heel stick blood samplings and their complications [2]. Current technology analyses the light reflected by the skin and the subcutaneous tissue providing a useful alternative measurement for total serum bilirubin (TSB). Neonatal skin colour and thickness represents important variables in TcB measurements. Different devices have been individually validated versus spectrophotometric or high pressure liquid chromatography determination of TSB, often on neonates with a single ethnic background [3].

To provide useful clinical information, we compared the performance of the three most widespread transcutaneous bilirubinometers on a multiracial population of term and late pre-term neonates.

## Methods

Infants born at the well baby nursery of the University “Federico II” of Naples with gestational age over 35 weeks were enrolled between January and December 2009. The investigation protocol was approved by the local ethical committee and parental consent was obtained.

Infants with Rh or ABO isoimmunisation, major congenital malformations, haemoglobinopathies or evidence of liver disease were excluded from the study.

Infants with post-natal age from 4 to 75 hours were measured TSB by an experienced resident (SL) as ordered by the attending clinician unaware of the study purpose. Samples were obtained from heelsticks in capillary tubes, centrifuged at 3000 rpm for five minutes and then read by a direct spectrophotometer (GB13A, Bertocchi Elettromedicali, Cremona, Italy). Spectrophotometry was used as a reference as it is the technique used in daily routine and has better agreement with high pressure liquid chromatography (HPLC) than any other technique [4].

Within 20 minutes before TSB determination, TcB was measured on the infant’s forehead, protected from direct sunlight and avoiding areas with hair, bruises, nevi or other skin anomalies.

We used three well known transcutaneous bilirubinometers according to the manifacturer’s instructions. Bilicheck (SpectRX, Norcross, GA) was calibrated using a Bilical before each measurement, calculated averaging five readings. The device scans the whole spectrum of visible light and automatically subtracts the light reflected by confounding factors like haemoglobin or melanin. BiliMed (Medick, S.A. France) operates by ten light emitting diodes that form a single beam when hitting the newborn skin. A silicon diode captures the reflected light and the internal software calculates the highest intensity for every wavelength range. BiliMed was used as recommended at 2 cm from the neonate’s forehead. JM-103 (Draeger Medical Systems, Inc., Telford, PA) uses 2 wavelengths and a dual optical path system; each measurement was calculated averaging five readings.

All measurements were performed in the ambient morning light of the nursery, in random order according to a computer generated randomization table.

Data were analyzed using MedCalc software v.11 (MedCalc Software, Mariakerke, Belgium). Linear regression analysis, Pearson correlation coefficients, Bland-Altman plots and receiver operating curve (ROC) were performed.

## Results

A total of 343 determinations obtained on 253 Caucasian and 36 West African infants were included in the study. The demographic characteristics are shown in Table 1.

**Table 1:** Basic characteristic and clinical variables of infants with hyperbilirubinemia

**Mean birthweight (gr)**	**3060**
range	1800-4350
Gestational age (week range)	35-41
Male sex	119
Famale sex	170
Ethnic group	
Caucasians	253
non-Caucasians	36
Total measurement	343
Mean TSB (mg/dl)	9.5
range	(15.8-0.7)
Mean TcB Bilicheck (mg/dl)	9.8
range	(17.2-0.4)
Mean TcB Minolta JM103 (mg/dl)	9.8
range	(18.8-0.1)
Mean TcB Bilimed (mg/dl)	10.4
range	(17.5-2.9)

**Figure 1:**
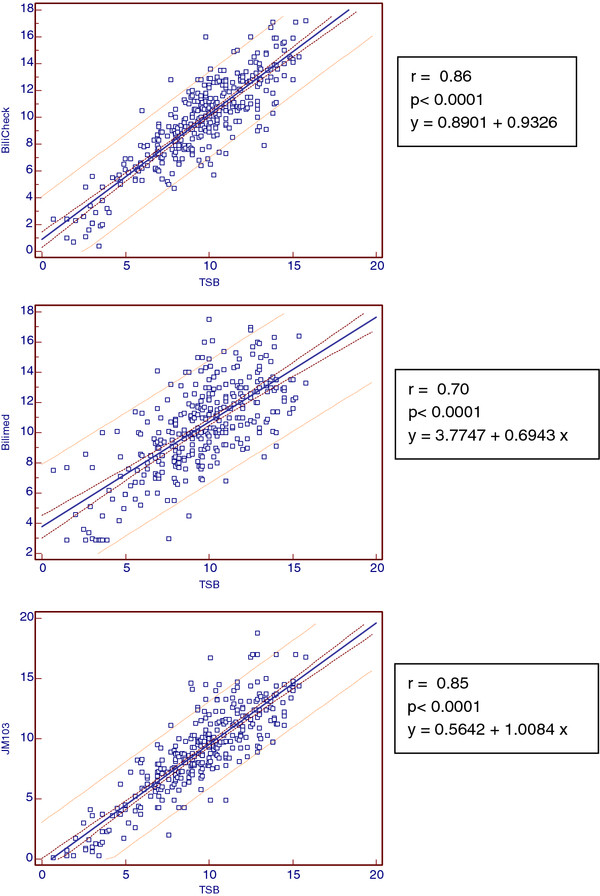
**A. Pearson’s correlation coefficients for the whole population.B**. Pearson’s correlation coefficients for African population.

TSB was determined over a wide concentration range (15,8 – 0,7 mg/dl) with a mean of 9.5 mg/dl. Related TcB values using Bilicheck (TcB-BC), BiliMed (TcB-BM), and JM-103 (TcB-JM) are reported in Table 1. Figure 1A shows the linear regression analysis for the three transcutaneous bilirubinometers on the whole study population. Pearson’s coefficients were almost equivalent for BiliCheck (0.86) and JM-103 (0.85) but substantially lower for BiliMed (0.70). Extrapolating non-Caucasian infants, correlation coefficients were 0.92 for JM-103, 0.88 for BiliCheck and 0.74 for BiliMed (Figure 1B).

**Figure 2:**
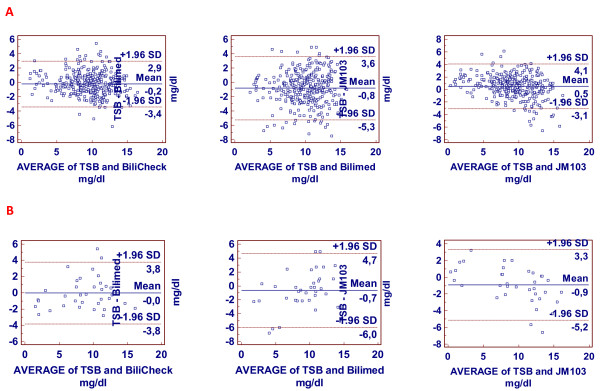
**A and 2B. Bland Altman plot for the whole population and for African neonates respectively.** These graphs show the mean difference between TSB and TcB for each transcutaneous bilirubinometer ± 1.96 SD that are index of imprecision of the instruments.

**Figure 3:**
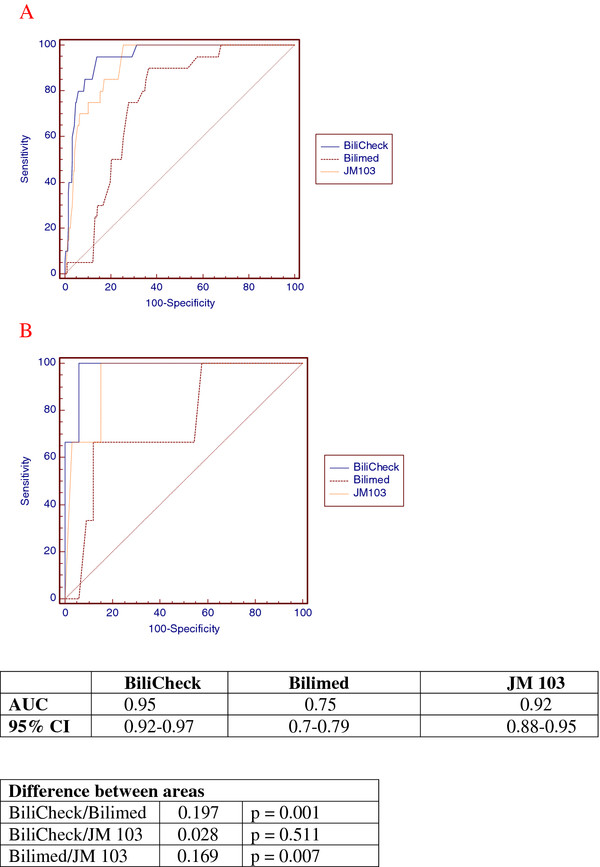
**ROC curves when TSB > 14 mg/dl was the outcome of choice for the three transcutaneous bilirubinometer (A: whole population; B: African neonates).** AUC and difference between areas are reported for whole population.

Error plots for the three devices are shown in Figure 2. Bilicheck showed a lower trend to underestimate TSB; this was more evident for BiliMed which also was the least accurate, while JM-103 had a tendency to overestimate and a distribution of 98% of measurements that was intermediate between Bilicheck and BiliMed. Figure 3 reports ROC curves for the three transcutaneous bilirubinometers when TSB > 14 mg/dl was used as cut-off, on the whole population (Figure 3A) and on the non-Caucasian subgroup (Figure 3B). The areas under the curve resulted 0.95 for Bilicheck, 0.75 for Bilimed and 0.91 for JM-103 on the whole population; for the non-Caucasian subgroup the areas under the curve were 0.98 for Bilicheck, 0.75 for Bilimed and 0.94 for JM-103.

## Discussion

In a comparative evaluation of the three most common transcutaneous bilirubinometers we showed that Bilicheck and JM-103 but not BiliMed are reliable screening tools for moderate neonatal hyperbilirubinemia. The latter device resulted inferior to Bilicheck in previous studies on Caucasian populations [5,6]. We found that on non-Caucasians BiliMed looses further ground to JM-103 and Bilicheck, the two transcutaneous bilirubinometers with the largest literature and broadest distribution. We also provided the first direct comparison of Bilicheck and JM-103 on a multiracial population as previous authors investigated these technologies on Asian or Caucasian infants [7-9].

In the present study, both JM-103 and Bilicheck resulted equally accurate screening tools over the range of concentrations tested, regardless of the skin pigmentation. JM-103, in particular, represents an improvement of a previous device. In the study of Robertson et al. JM-102 was less accurate than Bilicheck and influenced by the skin color. In the present study, JM-103 had a good performance on dark skinned infants, required no disposables to calibrate and was quicker to operate than Bilicheck.

Looking at the Bland Altman plots, the three devices had a tendency to overestimation on higher end of the tested concentration range. Although this may be *per se* protective against the clinical damage of hyperbilirubinemia, one would expect that newer technologies should be more accurate therefore expanding the concentration range where a TSB determination can be spared. We acknowledge a limitation of the study as we do not provide comparative data on preterm infants. Previous studies have shown that transcutaneous bilirubin evaluation can be useful on this population which is at increased risk of clinical damage as early as 24 weeks of gestational age [10]. It will be interesting to compare data on preterm infants since, as in the present study on late preterm and term infants, devices using different technologies may have variable performances. Clinicians need to be aware of these differences.

## Conclusions

In our multiracial neonatal population Bilicheck and JM-103, but not BiliMed, are equally reliable screening tools for hyperbilirubinemia.

## Competing interests

The authors have no competing financial interest in relation to the work described. no external financial support was provided for the study.

## Authors’ contributions

FR conceived the study and participated in its design and coordination; SL operated the bilirubinometers; FL, MS, ACB assisted with data collection and statistics; RM, PM, LC drafted the manuscript. All authors read and approved the final manuscrpit.

## Pre-publication history

The pre-publication history for this paper can be accessed here:

http://www.biomedcentral.com/1471-2431/12/70/prepub
